# Performance Comparisons of Youth Weightlifters as a Function of Age Group and Sex

**DOI:** 10.3390/jfmk6030057

**Published:** 2021-06-23

**Authors:** Satoshi Mizuguchi, Aaron J. Cunanan, Dylan G. Suarez, William E. Cedar, Mark A. South, Daniel Gahreman, William G. Hornsby, Michael H. Stone

**Affiliations:** 1 Center of Excellence for Sport Science and Coach Education, Department of Sport, Exercise, Recreation, and Kinesiology, East Tennessee State University, Johnson City, TN 37614, USA; cunanan@mail.etsu.edu (A.J.C.); suarezd@mail.etsu.edu (D.G.S.); southma@etsu.edu (M.A.S.); stonem@mail.etsu.edu (M.H.S.); 2 Olympic Sports Strength and Conditioning, Clemson University, Clemson, SC 29634, USA; wcedar@clemson.edu; 3 College of Health and Human Sciences, Charles Darwin University, Darwin 0815, Australia; daniel.gahreman@cdu.edu.au; 4 Department of Coaching and Teaching Studies, College of Physical Activity and Sport Sciences, West Virginia University, Morgantown, WV 26505, USA; william.hornsby@mail.wvu.edu

**Keywords:** youth, weightlifting, countermovement jump

## Abstract

This study was designed to provide an overview of weightlifting performance as a function of age group and sex and evaluate the potential of countermovement jump height (CMJH) as a tool to gauge performance potential. Data from 130 youth athletes (female, *n* = 65 & male, *n* = 65) were used to examine progression of performance (Total and Sinclair total) and the relationship between CMJH and Sinclair total while considering interactions between CMJH and age and/or sex. ANOVAs with post hoc analyses revealed that both totals had a statistical first-order polynomial interaction effect between age group and sex and the difference between age groups of 12–13 and 14–15 years old was statistically greater for male than female. A linear model, developed to examine the relationship, revealed that CMJH and CMJH x sex x age rejected the null hypothesis. Our primary findings are that male youth weightlifters have a higher rate of performance progression, possibly owing to puberty, and CMJH may be a better gauging tool for older male youth weightlifters.

## 1. Introduction

There appears to be a general consensus that introduction to sports in some form at a young age is beneficial from the standpoint of developing elite performance [1]. The recent related literature suggests that the development of elite performance may be best accomplished with general participation in a variety of sports early on and gradually narrowing down to a single sport [2]. Besides a general understanding of a path to elite performance, it is important to understand what a youth athlete needs in order to be able to reach elite performance in a specific sport [3]. This is because it is unlikely that every youth athlete will reach the elite level simply by following a suggested general path.

In the athlete talent identification literature, weightlifting is often grouped together with other sports whose performance is measured in centimeters, grams, or seconds, often abbreviated as cgs sports [2,4]. In the related literature, researchers generally agree that elite performance in cgs sports is more commonly achieved by early diversification and late specialization [2,4]. However, the literature appears to lack information on the development of youth athletes specifically for weightlifting, particularly as it corresponds to chronological age, which is used to set up levels of competition (i.e., age group). Our understanding of the development of youth weightlifters appears to be primarily that performance indicated by weight lifted shows a general increasing trend [5,6] and male weightlifters typically begin to out-perform female counterparts around the age of 14 [7]. Possible timings of performance benchmarks (e.g., weight lifted: body mass = 1) and variations in the rate of performance change have also been observed at least in female weightlifters [6]. Besides our current understanding, practitioners can further benefit from improved knowledge of performance development with respect to chronological age group and sex. Thus, such knowledge can help coaches to anticipate upcoming changes in performance and better plan the path towards a higher level of competition for each youth weightlifter.

In addition to enhanced understanding of performance development, a practical tool to gauge the current performance potential of a youth weightlifter can be useful. There has been evidence that higher-caliber weightlifters are likely to jump higher than lower-caliber counterparts [8,9]. Indeed, exercises frequently used by weightlifters in training are known to enhance vertical jump height and weightlifting performance [10]. The relationship between weightlifting and jumping performance is primarily supported by evidence derived from adolescent and young adult athletes [8,9,10]. Moreover, the available evidence indicates that some of the factors that also lead to a higher-caliber weightlifter, such as maximum strength, are likely those that lead to a better jumper [11,12]. For example, maximum strength is often thought to limit one’s force production capacity in a dynamic explosive movement, consequently limiting displacement of an object (e.g., body mass and/or barbell) [13]. Thus, vertical jump height has potential to be a practical tool to evaluate performance potential, not to mention the ease of measurement.

Given the potential benefits of improving the understanding of the sport of weightlifting, this study was designed with two goals. One was to help improve weightlifting coaches’ knowledge of a typical performance trajectory of a youth weightlifter given his or her age group and sex. The other was to contribute to the potential enhancement of performance monitoring through the use of vertical jump height. In order to meet our goals, this study was conducted with two objectives. One objective was to examine the progression of weightlifting total (both actual and Sinclair-adjusted) during the youth period in relation to operationally defined age groups and sex. The second objective was to examine the degree to which countermovement jump height (CMJH) reflects a youth weightlifter’s Sinclair total while considering age and sex. We hypothesized that weightlifting total would increase as a function of older age group with a possible bias towards male weightlifters due to androgens [14,15]. In addition, we hypothesized that CMJH and Sinclair total would be correlated possibly better for male weightlifters [12] but were unable to form a hypothesis on whether the correlation would be influenced by age due to the lack of pertinent literature and anecdotal observations.

## 2. Materials and Methods

### 2.1. Experimental Approach to the Problem

We conducted a retrospective analysis of publicly available data. We used CMJH data collected at the 2018 USAW Youth National Championships as part of the organization’s effort for talent identification. The data were publicly available and obtained from https://www.sportscienceed.com/blog/weightlifting-talent-identification (accessed on 14 June 2018). In addition, we used the publicly available results of the competitions (body mass, sex, age, best total, and best Sinclair total) at https://drive.google.com/drive/folders/1_wFI5q350rrjeWe0sz6WYtO_ulgVO064 (accessed on 14 June 2018). While medals are awarded to the top three places in each lift, the overall placements are determined by the total of the heaviest successful Snatch and Clean & Jerk, referred to as Total in this paper. Sinclair total is used to determine the best lifters of a competition by USAW and in an attempt to obviate the effect of body mass when comparing totals of lifters from different weight classes in the sport. Although the Sinclair formula is best intended for weightlifters in their late teens to early thirties, the International Weightlifting Federation suggests its use as a guideline for youth weightlifters [16].

### 2.2. Subjects

The publicly available CMJH and competition data were obtained for 130 youth weightlifters who competed in the 2018 USAW Youth National Championships (Table 1). The inclusion criteria were (1) participation and successfully scoring a total in the competition and (2) participation in the jump testing. To examine the effects of competition level based on age groups, the weightlifters were divided into four groups: 11 and under (U11), 12 to 13 (12–13), 14 to 15 (14–15), and 16 to 17 (16–17) years old. This study was approved as non-human subject research by the internal review board of East Tennessee State University.

### 2.3. Standardized Warm-Up

The standardized warm-up protocol consisted of 10 body-weight squats, 10 walking lunges (5 on each side), and 10 standing presses behind the neck with a PVC pipe. The rest period between each exercise ranged from approximately 15 to 30 s.

### 2.4. Countermovement Jump Test

CMJH was collected as the average of the two best trials (the best trials based on CMJH). The jumps were performed with a PVC pipe held on the back of the shoulders as in a back squat. Each trial began from the complete elect position. Each weightlifter was instructed to make the maximal effort for each jump and asked to perform a minimum of two maximal jumps from a self-selected depth. A switch mat (Just Jump System, Probotics Inc., Huntsville, AL, USA) was used to estimate jump height. This device has previously been reported to provide an overestimated jump height compared to a jump height calculated from flight time from a force plate. However, jump heights from the two methods likely share over 99% of their variances in common with no visible heteroscedasticity, an intraclass correlation coefficient (ICC) of 0.96, and a coefficient of variation of 3.7% [17]. In order to allow for comparison to flight-time-derived jump height using a force plate, the measured jump height was used to predict what would have been flight-time-derived jump height using a force plate (Table 1) [17]. Estimated 95% prediction intervals suggested a prediction error of ±1.28 cm and ±1.17 cm for maximum and minimum values, respectively, in our data set. Finally, for our data set, the ICC (two-way mixed effects with absolute agreement) was found to be 0.983 and the standard error of estimate was found to be 1.36 cm.

### 2.5. Statistical Analyses

To examine the progression of Total and Sinclair total over the youth period, a 4 × 2 between-subject analysis of variance (ANOVA) was conducted with each type of total being the dependent variable. The first four-level factor was age group, defined as U11, 12–13, 14–15, and 16–17. The second two-level factor was sex (female and male). Following the omnibus ANOVAs, various post hoc analyses were performed. These included trend analyses, interaction contrasts, and simple comparisons. Scheffe adjustment to the critical F score at the alpha level of 0.05 (from 2.68 to 8.04) was performed to control for an inflated type I error rate. When heteroscedasticity was suspected, heteroscedastic consistent covariance matrix 4 [18] was used to correct standard errors. Violations of any other assumptions of the general linear model were not suspected. Cohen’s d was calculated for all simple comparisons. Hopkins [19] suggests the following scale for interpretation: 0–0.2 for trivial, 0.2–0.6 for small, 0.6–1.2 for moderate, 1.2–2.0 for large, 2.0–4.0 for very large, and >0.4 for nearly perfect.

To examine the relationship between CMJH and Sinclair total and whether the relationship depends on age and/or sex, a linear model was developed with Sinclair total being the dependent variable. The independent variables were CMJH and its interaction with age and/or sex. Sex was converted to a dummy variable (female = 0 and male = 1). All the other variables were centered to avoid multicollinearity.

For all analyses, the data were screened for the assumptions of the general linear model first. When any of the assumptions failed, appropriate action was taken to ensure the accuracy of all modelling outcomes. The ANOVAs and their post hoc analyses were performed using SPSS ver. 25 (IBM Corp., released 2017, Armonk, NY, USA) while the rest of the analyses were performed with R (R Foundation for Statistical Computing, Vienna, Austria). The following functions were used for modelling: lm, gls, boot, and boot.ci. All null hypothesis testing for parameter estimates was performed with the critical alpha of 0.05.

## 3. Results

### 3.1. Development Overview

**Figure 1:**
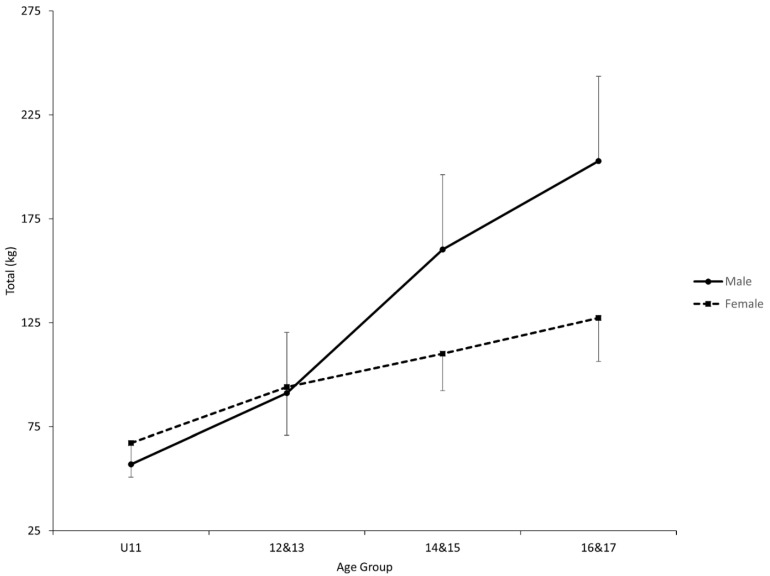
Comparisons of total between age groups and sexes. U11 = 11 and under. 12–13 = age group of 12 and 13-year-olds. 14–15 = age group of 14 and 15-year-olds. 16–17 = age group of 16 and 17-year-olds. The error bars indicate standard deviations. A statistically significant interaction effect was found for sex by age group overall. A statistically significant first-order polynomial trend was found for male and female. A statistically significant interaction contrast between male and female was found from 12–13 to 14–15. Statistically significant simple comparisons were found between U11 and 12–13, 12–13, and 14 & 15, and between 14–15 and 16–17, for male, and between U11 and 12–13 for female. Statistically significant simple comparisons between male and female were found for 14–15 and 16–17.

**Figure 2:**
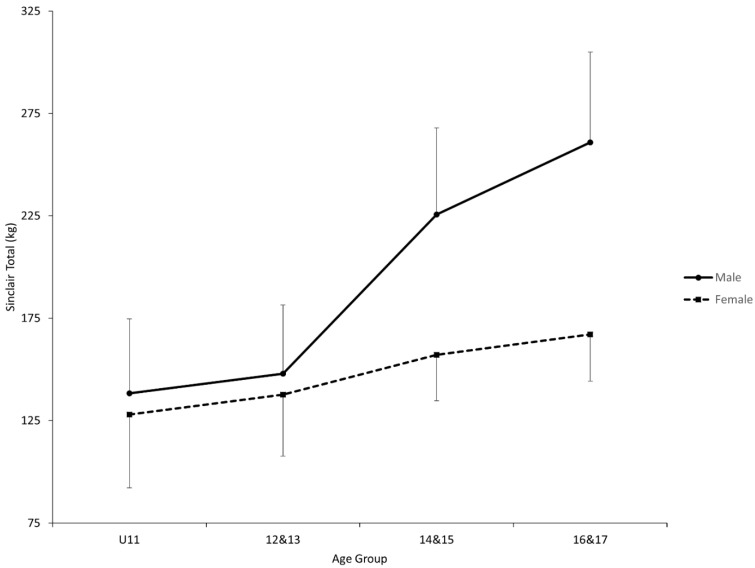
Comparisons of Sinclair total between age groups and sexes. U11 = 11 and under. 12–13 = age group of 12 and 13-year-olds. 14–15 = age group of 14 and 15-year-olds. 16–17 = age group of 16 and 17-year-olds. The error bars indicate standard deviations. A statistically significant interaction effect was found for sex by age group overall. A statistically significant first-order polynomial trend was found for male and female. A statistically significant simple comparison was found between 12–13 and 14–15 for male. A statistical simple comparison between male and female for 16–17.

Both ANOVAs showed a statistical interaction effect between age group and sex (Total, *p* < 0.0001 and F_(3, 122)_ = 11.36; Sinclair total, *p* < 0.0001 & F_(3, 122)_ = 20.84) in addition to the two statistical main effects for Total (Sex, *p* < 0.0001 and F_(1, 122)_ = 37.49; Age group, *p* < 0.0001 and F_(3, 122)_ = 109.06) and Sinclair Total (Sex, *p* < 0.0001 and F_(1, 122)_ = 42.17; Age group, *p* < 0.0001 and F_(3, 122)_ = 35.28) (Figure 1 and Figure 2, Table 2). For both dependent variables, each sex showed a statistical post hoc first-order trend (F_(3, 122)_ = 15.58 to 217.89). Furthermore, the first-order trends were statistically different between the sexes (Total, F_(3, 122)_ = 60.03; Sinclair total, F_(3, 122)_ = 18.88). A statistical post hoc interaction contrast was observed for both dependent variables between the age groups of 12–13 and 14–15 when comparing the sexes (Total, F_(1, 122)_ = 15.70; Sinclair total, F_(1, 122)_ = 12.95). Finally, there were no sex differences observed until the age group of 14–15 in both dependent variables (Total, F_(1, 122)_ = 25.21 and 51.31; Sinclair, F_(1, 122)_ = 33.34 and 67.88).

While the above-mentioned observations were identical between the two dependent variables, there were differences between the two. The first difference was that male weightlifters showed a statistical post hoc simple comparison within each pair of two adjacent age groups for Total (F_(1, 122)_ = 10.43 to 41.50) while the only statistical post hoc simple comparison for Sinclair Total was between the age groups of 12–13 and 14–15 (F_(1, 122)_ = 38.69) (Table 3). The second difference was that, for female weightlifters, only the post hoc simple comparison between the age groups of U11 and 12–13 rejected the null hypothesis for Total among the three pairs of adjacent age groups (F_(1, 122)_ = 10.76) while no post hoc simple comparisons rejected the null hypothesis for Sinclair Total.

### 3.2. Jump Height as a Monitoring Tool

The first attempt at a linear model was suspected for heteroscedasticity, dependence of error, and influential cases. Thus, another linear model was developed using the technique of generalized least squares while accounting for heteroscedasticity and dependence of error. Bootstrapping was then applied to minimize the effect of influential cases [20,21]. A 95% confidence interval was developed for each parameter estimate using the percentile method after bootstrapping (Table 4). Of the three interaction terms, only CMJH × Age × Sex had its coefficient rejecting the null hypothesis.

## 4. Discussion

This study was conducted to provide further information for weightlifting coaches with respect to the development of weightlifting total as a function of age group and sex and the potential of CMJH to be used for gauging such development. The results of this study appear to support the following findings to various degrees. Male weightlifters generally experience a steep increase in weightlifting total between the age groups of 12–13 and 14–15, which is unlikely to occur in female weightlifters. This increase in male weightlifters appears to be caused by factors besides an increase in body mass (i.e., muscle mass), unlike in the other age groups. Prior to the age group of 14–15, male and female weightlifters generally perform similarly to each other relative to their age group. Both sexes generally experience an overall increase in weightlifting total over the examined age groups. However, male weightlifters experience a greater rate of improvement overall. Some, but not all, of the overall increase is likely related to an increase in body mass (i.e., muscle mass). CMJH appears to be a general indicator for a youth weightlifter’s Sinclair total. However, it is possibly a more effective indicator of weightlifting performance for male weightlifters with increasing age. Our findings appear to support both of our hypotheses regarding the progression of weightlifting total across age groups and the relationship between CMJH and Sinclair total.

In youth weightlifters, our results are in support of an overall increase that is generally expected in both Total and Sinclair total for both sexes (Figure 1 and Figure 2). This finding is supported by the statistical first-order trend for each sex. It is a well-documented phenomenon that physical performance increases as a child matures [22,23,24], although our results are not able to eliminate training age as a contributing factor to the overall increase. However, closer examination of our results suggests that the overall increase is expected to be generally greater for male weightlifters, as supported by the statistical first-order interaction. As a result, male weightlifters are likely to produce a greater Total and Sinclair total between the age groups of 14–15 and 16–17 than female counterparts, as supported by the statistical simple comparisons (male weightlifters had greater Sinclair total in our sample also in the first two age groups). The difference in the rate of increase is likely due to a sudden change in both totals among male weightlifters some time between the age groups of 12–13 and 14–15, as supported by the statistical interaction contrast and the lack thereof in the other age periods.

The sudden increase in the difference between the two sexes, however, might be explained based on the related literature of human development. During puberty, a steep rise in androgen concentrations takes place among male children, which in turn leads to greater enhancement of fitness qualities compared to female counterparts [14,15]. In fact, the sudden increase in the performance discrepancy has also been observed in other studies examining various performance measures among children of similar ages [15,22,24,25] as well as for Total [7,26]. In particular, Huebner et al. [7] reported the likely onset of the sudden increase in Total to occur around the age of 14.

Our results support the possibility that the sudden rise is at least partially independent of body mass changes in male youth weightlifters between the age groups of 12–13 and 14–15. This is because the sudden rise is expected in Total and Sinclair total, which attempts to obviate body mass differences [16]. While our results cannot provide direct evidence for what other factors besides body mass change could be contributing, potential contributors are the effects of testosterone on force production and adaptations to resistance training. Specifically, higher testosterone concentrations may be related to superior performance in squatting, sprinting, and jumping [27,28]. In addition, individuals with higher testosterone concentrations may exhibit a greater magnitude of adaptation to resistance training [29,30,31].

As a whole, maturation and growth, part of which occurs during puberty, are generally known to lead to improvements in fitness qualities [22,24,32,33]. For example, Pena-Gonzalez et al. [32] observed statistical differences between three maturation level groups of male youth soccer players without prior structured resistance training in various fitness tests, such as half-squat one-repetition maximum, 30 m sprint time, and agility T-test time. Furthermore, these investigators and others observed varying effects of physical fitness training (e.g., strength, plyometric, and sprint training) according to different maturation levels [32,34,35,36,37]. In other words, not only can maturation and growth alone influence one’s ability to lift more weight but they also can impact the effectiveness of physical fitness training. In contrast to fitness qualities, technical coordination does not appear to be impacted by maturation level, at least among youth soccer players [38,39], although older youth soccer players and longitudinally tracked youth soccer players appear to perform better in soccer-related skill tests speculatively due to the accumulation of more practice [33,40]. However, with the lack of empirical evidence for or against a possible role of strength in weightlifting technical execution with a high load, the effects of maturation and growth on technical development should not be ruled out completely.

In contrast to the similar observations already discussed for Total and Sinclair total, there was one notable difference—the disappearance of all statistical simple comparisons except the one between the age groups of 12–13 and 14–15 for male weightlifters once the Sinclair formula was applied. This implies a possibility that the retained difference was due to an improvement in the force-producing ability independent of muscle mass, while the vanished differences mainly were attributed to an increase in body mass (i.e., muscle) in the sample. While improvement from one age group to the next may be inferred to be largely due to muscle mass change based on this observation, it is not clear whether a development trend in both totals would appear similarly to the one observed in this study if youth weightlifters were tracked longitudinally. As already discussed, hormonal changes, particularly changes in androgen concentrations, can positively influence muscle force production independent of muscle mass changes [27,28]. Thus, caution should be exercised when evaluating the value of the efficacy of technical practice and physical fitness training from an age group to the next (i.e., it is still possible for technical practice and physical fitness training to make a substantial contribution to an increase in weightlifting total from an age group to the next).

Finally, it may be worth noting that female weightlifters showed only one statistical simple comparison in this sample. It is possible that this observation was caused by sampling error and/or the difficulty associated with detecting smaller age group differences over larger individual differences. At the same time, this observation can be explained by the fact that, while male youth weightlifters benefit from increased muscle mass and reduced fat mass due to increased androgen secretion, little of the same alteration is available to female counterparts [41]. The lack of statistical simple comparisons after the age group of 12–13 in female weightlifters may be related to a smaller amount of gain of related fitness qualities due to genetic or hormonal differences. This explanation also supports the finding that female youth weightlifters generally have a lower rate of overall increase in Total and Sinclair total.

Our modelling results suggest CMJH as a likely indicator of a youth weightlifter’s Sinclair total (Table 4). Based on our results, on average, an increase of 1 cm in CMJH is likely to be related to an increase of 1.8 to 4.0 kg in Sinclair total. Other investigators have also observed a likely relationship between one repetition maximum of a weightlifting exercise and vertical jump height with different types of samples [8,42,43]. Moreover, our results suggest a likely dependency for the relationship that, for male youth weightlifters, increases of 1 cm in CMJH and 1 year in age further add 0.3 to 1.5 kg to their Sinclair total on average, while this additional benefit is not likely for female youth weightlifters. As a whole, it appears that CMJH is a more effective monitoring tool for Sinclair total in a male youth weightlifter, particularly as they age.

At the same time, our observation for CMJH can be due to sampling error caused possibly by the varying competitive levels of samples at each age (e.g., some ages had less competitive weightlifters than the others) (Table 1). On the other hand, different effects of sex hormones on performance potential during and after puberty may once again explain why the effectiveness improves for male youth weightlifters. Androgens are understood to have major impacts on a wide range of fitness qualities [15,41]. Some of these qualities (e.g., strength, explosiveness, and muscle mass) are thought to have impacts on both weightlifting performance and jump height [11,12,43,44,45]. In addition, because estrogens promote deposition of adipose tissue, despite growth, it is possible that female youth weightlifters make less progress in the related fitness qualities during and after puberty than before puberty. In fact, previous investigations reported a likely increase in the gap in performance between male and female youth weightlifters starting around the age of 14 [7] as well as possible slowing or even plateauing of knee extension and flexion strength per body mass and vertical jump height in female children starting around the age of 12, the age at which female children on average reach the peak height velocity [32], likely reflecting the highest rate of physical change [22,24].

## 5. Conclusions

In conclusion, it appears reasonable to summarize our findings as follows. A greater rate of increase is expected in weightlifting performance for male youth weightlifters particularly between the age groups of 12–13 and 14–15, although both sexes show a trend of increase. While the effectiveness may vary between sexes and ages, CMJH can be used as a general indicator of a youth weightlifter’s possible Sinclair total. These findings appear to be supported by the results of our study as well as previous studies in the related literature. However, there are three major points that warrant further investigation. The first is the actual rate of increase in both totals during the youth period (i.e., longitudinal study). While our results do provide estimates of age-group-related differences, some of these estimates are also inherently influenced by individual differences to some degree. Thus, future studies that longitudinally follow youth weightlifters are needed to provide more accurate estimates. The second point is the age and sex dependency of the relationship between CMJH and Sinclair total. Our results do leave some uncertainty on this point given possible sampling error due to varying competitiveness between sexes and ages, although our understanding of human development provides logical explanations for our observations. Future studies should consider ensuring that samples at each age represent high to low placing weightlifters. The third is that our findings are strictly based on youth weightlifters’ chronological age, which is used by USA weightlifting to hold competitions. Thus, a similar study may be repeated using maturation level rather than chronological age to evaluate the progress of performance. Finally, it is important to note that possible training age effects are not obviated from our results in order to provide more realistic estimates of performance change for practitioners.

### Practical Implications

It is important to emphasize that our practical implications are based on chronological age and not necessarily maturation level as chronological age is used to set up competition levels. The following are take-home points for weightlifting coaches. (1) A coach should expect a difference in the rate of improvement between male and female youth weightlifters, particularly between the age groups of 12–13 and 14–15. (2) Given the lower overall rate of improvement in fitness qualities, a female youth weightlifter may benefit more from a greater emphasis on muscular and strength development at an earlier age. (3) As a general rule, an increase in CMJH suggests a greater possibility of an increase in Sinclair total, with a male and older youth weightlifter likely showing a greater increase for a given increase in CMJH. As performed in this study, a device such as a switch mat can be readily used to conduct periodical assessments of CMJH.

## Figures and Tables

**Table 1:** Characteristics of participating youth weightlifters.

Sex	Age Group	Age	Placement	Total (kg)	Sinclair (kg)	BM (kg)	CMJH (cm)	Pre CMJH (cm)
Female (*n* = 65)	11 and under	9 (*n* = 1)	12.0	65.0	118.3	40.76	33.27	22.44
	(*n* = 13)	10 (*n* = 5)	7.4 ± 4.3	59.2 ± 14.7	126.1 ± 38.9	29.74 ± 20.35	32.92 ± 5.12	22.13 ± 4.48
		11 (*n* = 7)	8.3 ± 5.9	73.1 ± 17.4	130.5 ± 38.8	41.01 ± 25.52	35.45 ± 7.17	24.35 ± 6.27
	12–13	12 (*n* = 1)	5.0	75.0	129.8	43.17	38.86	27.33
	(*n* = 12)	13 (*n* = 11)	5.6 ± 5.3	95.8 ± 23.5	138.3 ± 31.3	56.91 ± 13.80	38.01 ± 5.58	26.59 ± 4.88
	14–15	14 (*n* = 8)	9.0 ± 6.1	107.8 ± 22.6	153.9 ± 27.7	36.64 ± 34.40	38.96 ± 5.14	27.42 ± 4.50
	(*n* = 17)	15 (*n* = 9)	9.9 ± 6.3	112.1 ± 13.1	159.8 ± 17.7	48.86 ± 19.15	40.41 ± 4.53	28.69 ± 3.96
	16–17	16 (*n* = 10)	6.9 ± 4.5	128.6 ± 27.5	173.6 ± 26.1	55.32 ± 21.65	42.06 ± 5.81	30.13 ± 5.09
	(*n* = 23)	17 (*n* = 13)	10.1 ± 5.6	126.2 ± 15.2	162.0 ± 19.6	49.66 ± 30.46	42.83 ± 6.02	30.80 ± 5.26
Male (*n* = 65)	11 and under	9 (*n* = 0)	N/A	N/A	N/A	N/A	N/A	N/A
	(*n* = 6)	10 (*n* = 2)	10.0 ± 7.1	59.5 ± 7.8	153.9 ± 5.1	32.03 ± 4.71	34.04 ± 7.18	23.11 ± 6.28
		11 (*n* = 4)	10.3 ± 9.1	55.8 ± 12.7	130.3 ± 44.3	36.80 ± 6.59	36.45 ± 7.51	25.22 ± 6.57
	12–13	12 (*n* = 9)	9.8 ± 4.6	73.3 ± 20.7	146.2 ± 40.1	42.26 ± 9.73	37.62 ± 7.02	26.25 ± 6.14
	(*n* = 25)	13 (*n* = 16)	5.6 ± 3.3	101.1 ± 29.0	148.8 ± 30.7	66.15 ± 25.36	38.39 ± 9.56	26.92 ± 8.36
	14–15	14 (*n* = 8)	5.6 ± 3.3	141.3 ± 26.5	217.1 ± 45.8	61.49 ± 23.18	46.64 ± 8.89	34.14 ± 7.77
	(*n* = 16)	15 (*n* = 8)	4.5 ± 3.9	179.1 ± 35.5	234.1 ± 39.6	73.72 ± 18.95	53.82 ± 10.83	40.41 ± 9.47
	16–17	16 (*n* = 3)	9.0 ± 3.6	141.0 ± 16.6	214.2 ± 23.2	56.65 ± 2.94	65.28 ± 1.32	50.44 ± 1.15
	(*n* = 18)	17 (*n* = 15)	7.3 ± 4.6	215.1 ± 31.8	270.2 ± 41.5	79.41 ± 17.66	58.10 ± 7.85	44.16 ± 6.86

Mean ± Standard Deviation. Sinclair = Sinclair total, BM = body mass, CMJH = countermovement jump height, Pre. CMJH = predicted force plate CMJH (flight time) with prediction error of ±1.28 cm and ±1.17 cm for maximum and minimum values, respectively, in our data.

**Table 2:** Mean differences between age groups within a sex.

Sex	Comparison	Total				Sinclair Total			
		Mean Diff (kg)	95% CI			Mean Diff (kg)	95% CI		
Female	U11 vs. 12–13	−26.9	−43.2	to	−10.7	−9.7	−36.0	to	16.6
	12–13 vs. 14–15	−16.0	−31.9	to	−0.1	−19.5	−39.9	to	1.0
	14–15 vs. 16–17	−17.2	−29.3	to	−5.2	−10.0	−24.3	to	4.2
Male	U11 vs. 12–13	−34.1	−49.4	to	−18.9	−9.7	−46.6	to	27.2
	12–13 vs. 14–15	−69.1	−90.3	to	−47.8	−77.8	−102.5	to	−53.0
	14–15 vs. 16–17	−42.5	−68.6	to	−16.5	−35.2	−64.6	to	−5.9

U11 = 11 and under. Mean diff = mean difference. 95% CI = 95% confidence interval.

**Table 3:** Cohen’s d for simple comparisons.

			Female				Male			
Total			**U11**	**12–13**	**14–15**	**16–17**	**U11**	**12–13**	**14–15**	**16–17**
	Female	U11								
		12–13	1.31							
		14–15		0.75						
		16–17			0.90					
	Male	U11	0.73							
		12–13		0.12			2.01			
		14–15			1.75			2.06		
		16–17				2.25			1.11	
Sinclair total	Female	U11								
		12–13	0.29							
		14–15		0.71						
		16–17			0.44					
	Male	U11	0.25							
		12–13		0.33			0.24			
		14–15			2.01			1.99		
		16–17				2.29			0.82	

U11 = 11 and under. 12–13 = age group of 12 and 13-year-olds. 14–15 = age group of 14 and 15-year-olds. 16–17 = age group of 16 and 17-year-olds.

**Table 4:** General linear model summary.

Intercept *			CMJH *			CMJH × Age			CMJH × Sex			CMJH × Age × Sex *		
Coef	Bias	95% CI (LL:UL)	Coef	Bias	95% CI (LL:UL)	Coef	Bias	95% CI (LL:UL)	Coef	Bias	95% CI (LL:UL)	Coef	Bias	95% CI (LL:UL)
675.9	−509.2	160.6:172.9	4.8	−2.0	1.8:4.0	0.1	−0.5	−0.8:0.1	−0.4	1.5	−0.3:2.4	−0.4	1.3	0.3:1.5

Coef = Original estimate prior to bootstrapping, Bias = bias in an original estimate found after bootstrapping, 95% CI = 95% confidence interval using the percentile method. All estimates are in the original scales. An asterisk * indicates rejection of the null hypothesis.

## Data Availability

The data that support the findings of this study are available from the corresponding author upon reasonable request.
